# Chromosomal level genome assembly of medicinal plant *Chrysosplenium macrophyllum*

**DOI:** 10.1038/s41597-025-05546-z

**Published:** 2025-07-15

**Authors:** Niyan Xiang, Tao Yuan, Shuo Liu, Tiange Yang, Xing Liu, Rui Qin, Liu Hong

**Affiliations:** 1 Hubei Provincial Key Laboratory for Protection and Application of Special Plant Germplasm in Wuling Area of China, College of Life Sciences, South-Central Minzu University, Wuhan, 430074 China; 2 School of Ecology and Environment, Tibet University, Lhasa, 850000 China; 3 School of Resources and Environmental Science, Hubei University, Wuhan, 430062 China; 4 State Key Laboratory of Hybrid Rice, Laboratory of Plant Systematics and Evolutionary Biology, College of Life Sciences, Wuhan University, Wuhan, 430072 China

**Keywords:** Plant evolution, Population genetics

## Abstract

*Chrysosplenium macrophyllum* Oliv., a perennial herb native to China, is widely used in traditional medicine for its notable therapeutic properties. However, the absence of a reference genome has constrained its full potential for research and application. This study presents the first chromosome-level ***de novo*** genome assembly of *C. macrophyllum*, constructed by integrating long reads from Oxford Nanopore Technologies (ONT), short reads from BGI, and Hi-C data. The final assembly spans 2.55 Gb, with a scaffold N50 of 93.38 Mb, and 83.70% of the genome has been assigned to 22 chromosomes. The mapping rate of the BGI short reads to the genome is approximately 97.94%, and BUSCO analysis reveals that 97.94% of the predicted genes are complete. A total of 62,921 protein-coding genes were predicted, with functional annotations for 93.67% of them. This chromosome-level genome assembly represents an important resource for expanding our understanding of *Chrysosplenium* species and supports future genomic studies and applications.

## Background & Summary

*Chrysosplenium*, a genus of small perennial herbaceous plants, occupies a distinctive position within the Saxifragaceae family^1^. Currently, there are approximately 80 species of *Chrysosplenium* worldwide, with the majority found in Asia, Europe, and North America in the northern hemisphere and a few species occurring in temperate regions of the Southern Hemisphere^2–4^. These species predominantly thrive in shady and humid habitats at altitudes ranging from 450 to 4800 meters, including alpine meadows, alpine shrubs, and high gravel gaps^5^. China is recognized as one of the centers of diversity for *Chrysosplenium*, harboring around 40 species, 24 of which are endemic to the country^6^. They are primarily distributed in the southwestern, northern, and central regions of China, with a significant concentration in the provinces of Shaanxi, Sichuan, Yunnan, and Xizang^7^. The genus *Chrysosplenium*, rich in various compounds such as flavonoids and triterpenes, has high medicinal value duo to its wide-ranging pharmacological properties, including anti-tumor, antibacterial, antiviral, hepatoprotective, and insecticidal activities^8^.

**Fig. 1:**
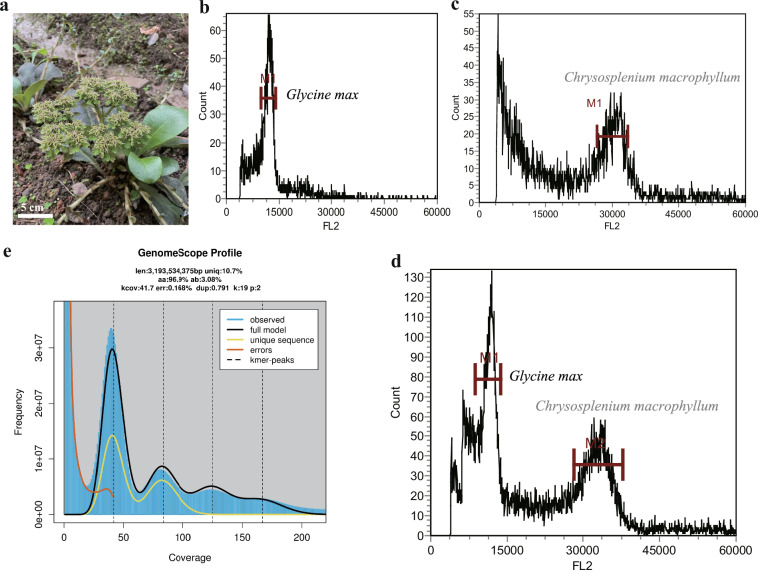
Genome assembly of *Chrysosplenium macrophyllum*. (**a**) Morphology of C. macrophyllum. (**b**). Flow cytometry histogram of Glycine max nuclei (FL2 signal, used as an internal reference). (**c**) Flow cytometry histogram of *C. macrophyllum* nuclei (FL2 signal). (**d**) Flow cytometry histogram of a mixed sample containing nuclei from both *G. max* and *C. macrophyllum* (FL2 signal), demonstrating clear peak separation for genome size estimation. (**e**) GenomeScope 2.0 profile of *C. macrophyllum* based on BGI short-read sequencing (k = 19), showing k-mer frequency distribution and estimated genome characteristics.

*Chrysosplenium macrophyllum* Oliv., is a perennial herbaceous plant belonging to the subgenus *Alternifolia*, and is a unique species native to China^9^ (Fig. 1a). Based on specimen records, it is mainly distributed in subtropical regions of China^10^. *C. macrophyllum* is a widely used folk herbal, traditionally employed in treating various ailments such as infantile convulsions, ecthyma, scalds, and lung and ear disorders^6^. While a pseudo-chromosome level genome for *Tiarella polyphylla* within Saxifragaceae family has been published, limited genomic information is available for species of the *Chrysosplenium* genus^11^. Previous research has predominantly focused on the chloroplast genome of *Chrysosplenium*, with no studies addressing its nuclear genome^12–16^. Furthermore, *C. macrophyllum* has received limited research attention, which has significantly hindered the development and utilization of its medicinal potential.

In this study, we have unveiled the whole-genome sequences of *C. macrophyllum* for the first time, achieved through the integration of Oxford Nanopore Technology (ONT) long reads, Beijing Genomics Institute (BGI) short reads, and high-throughput chromatin conformation capture sequencing (Hi-C) reads. The assembled genome size is approximately 2.55 Gb, with a scaffold N50 length of 93.38 Mb. Of the assembled sequences, 83.70% (2.14 Gb) were anchored to 22 pseudo-chromosomes. The genome contains 62,921 protein-coding genes, with annotations available for 93.67% of them. Additionally, we identified 316 miRNAs, 2,768 tRNAs, 2,348 rRNAs, and 1,467 snRNA. This newly assembled genome serves a crucial resource for investigating the evolutionary history of Saxifragaceae, studying the biosynthesis of bioactive compounds, and exploring its potential medicinal value as a Chinese endemic plant.

## Methods

### Plant materials

We collected samples from Qizimei Mountain National Nature Reserve for BGI sequencing, ONT sequencing, Hi-C sequencing, and transcriptome sequencing, as well as for flow cytometry analysis. Materials for chromosome karyotype analysis came from Saiwudang National Nature Reserve. All voucher specimens are stored in the Herbarium of South-Central Minzu University (HSN).

### Genome sequencing

To assemble and annotate the genome of *C. macrophyllum*, we combined short-read, long-read, Hi-C and transcriptome sequencing. Genomic DNA was extracted from young leaves of *C. macrophyllum* using a modified cetyltrimethylammonium bromide (CTAB) method^17^, and its concentration, purity, and integrity were assessed with a NanoDrop (NanoDrop Technologies, Wilmington, DE, USA) and a Qubit 3.0 fluorometer (Life Technologies, Carlsbad, CA, USA), and 0.75% agarose gel electrophoresis. A short-read library was prepared using the VAHTS Universal Plus DNA Library Prep Kit for MGI V2 (Vazyme Biotech Co., Ltd., Nanjing, China), followed by sequencing on the DNBSEQ-T7 platform (BGI Inc., Shenzhen, China), generating approximately 457.28 Gb of raw data, with an estimated genome coverage of 143×.

To complement the short-read data, long-read sequencing was performed using Oxford Nanopore Technologies (ONT). High molecular weight DNA was fragmented using a Megaruptor, and DNA fragments were selected and ligated to adapters using the Nanopore SQK-LSK109 kit. Sequencing on the PromethION platform produced 269.58 Gb of long-read data from approximately 3 million reads (N50 = 29 kb, longest read = 834 kb).

Hi-C sequencing was applied to further improve the assembly by capturing chromatin interactions. Hi-C libraries were constructed with a modified Belton *et al*.^18^ workflow. Chromatin was cross-linked, digested, and labeled, with interacting DNA fragments captured using streptavidin magnetic beads. The Hi-C libraries were sequenced on the DNBSEQ-T7 platform (BGI Inc., Shenzhen, China), generating 355 Gb of data, which were used to assist in the subsequent of pseudochromosomes.

For transcriptome sequencing, RNA was extracted from roots, stems, and leaves of plants from the same population used for genomic sequencing. These RNA samples were pooled in equal proportions, followed by library preparation and sequencing on both the DNBSEQ-T7 (BGI Inc., Shenzhen, China) and PromethION platforms (Oxford Nanopore Technologies, USA). This generated 6.12 Gb and 12.39 Gb of raw data, respectively, providing valuable data for the subsequent genome annotation.

All library preparation and sequencing were conducted by Wuhan Benagen Technology Co. Ltd. (Wuhan, China).

### Genome size estimation

Genome size was first measured by flow cytometry (Sysmex CyFlow® Cube6) at Jiyuan Biotech Co., Ltd (Guangzhou, China). A standard reference sample of *Glycine max* (Fig. 1b), with a known genome size, served as the benchmark. The analysis revealed that the genome size of *C. macrophyllum* is approximately 3.2 Gb (Fig. 1c,d). To further evaluate genome size, we carried out a k-mer–based genome survey. Raw BGI reads were quality-filtered with fastp v0.21.0^19^, which removed adapters, short fragments and low-quality bases. We then counted 19-mers frequencies with Jellyfish v2.2.10^20^ and assessed genome characteristics using GenomeScope v2.0^21^. The k-mer profile predicted a genome size of 3.19 Gb, a heterozygosity rate of 3.08%, and a duplication level of 89.26% (Fig. 1e).

### Karyotype analysis

Karyotype analysis of *C. macrophyllum* was conducted at OMIX Technologies Corporation (Chengdu, China) to identify chromosome number and ploidy. Active root tip meristematic tissues were obtained by culturing collected *C. macrophyllum* plants. Root tips, approximately 1.5–2 cm in length, were collected and exposed to a nitrous oxide environment to induce mitosis, thereby increasing the number of cells in the metaphase stage. These root tips were then diced, digested, and treated with a mixture of 1% pectolyase Y23 and 2% cellulase Onozuka R-10. Cells were subsequently gathered via centrifugation and resuspended in 90% acetic acid. A drop of the cell suspension was placed on a slide, which was kept in a box lined with moist paper. Chromosomes were stained with the fluorescent dye 4’,6-diamidino-2-phenylindole (DAPI). Metaphase cells with well-dispersed chromosomes were counted using an Olympus BX63 fluorescence microscope. Further confirmation of chromosome number and ploidy was achieved through fluorescence *in situ* hybridization (FISH), employing telomeric repeats Oligo-(TTTAGGG)6 as probes for chromosome counting and 5S rDNA repeats for ploidy determination. Observations were made using the Olympus BX63 fluorescence microscope.

**Fig. 2:**
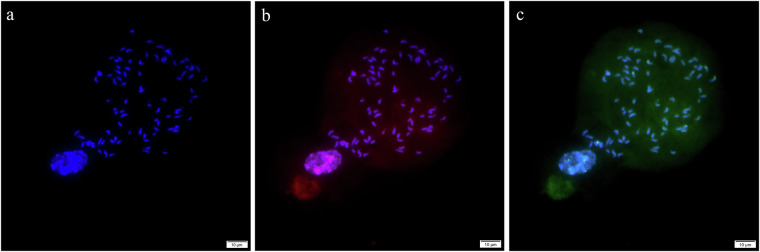
Chromosome counts and ploidy of *C. macrophyllum*. Fluorescent chromosome staining (**a**), telomere fluorescence *in situ* hybridization (**b**), and 5S rDNA fluorescence *in situ* hybridization (**c**) results of *C. macrophyllum*.

The karyotype analysis revealed that *C. macrophyllum* has a total of 88 chromosomes (Fig. 2a). FISH analysis using telomeric repeats probes showed clear fluorescent signals at the telomeres of various chromosomes, confirming the observed chromosome count of 88 (Fig. 2b). Additionally, FISH analysis with 5S rDNA repeat probes revealed that all cells in the sample exhibited 8 hybridization signals (Fig. 2c). Based on these findings, *C. macrophyllum* is confirmed to be an octoploid species with a chromosomal configuration of 2n = 8x = 88.

### De novo genome assembly

**Fig. 3:**
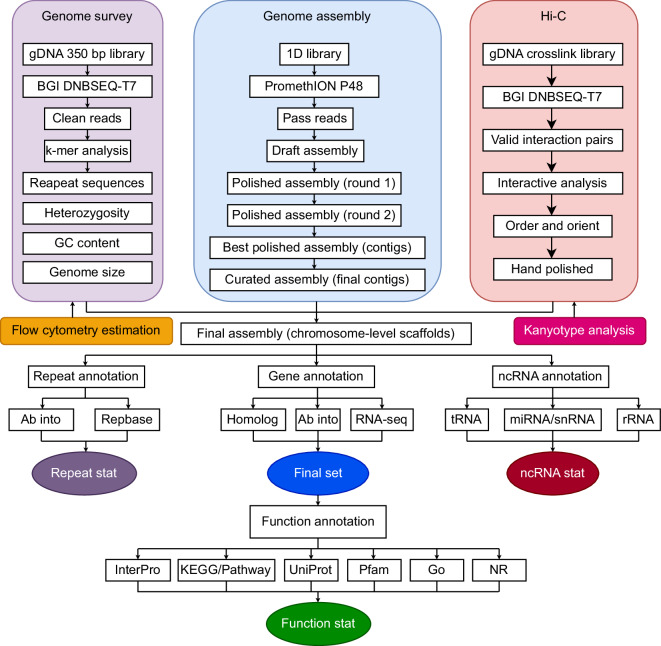
The pipelines overview of *C. macrophyllum* chromosome-level genome assembly and annotation.

The pipeline for the *C. macrophyllum* chromosome-level genome assembly and annotation is illustrated in Fig. 3. To assembly the contigs, low-quality Nanopore raw reads with a quality score below 7 were filtered out using Oxford Nanopore GUPPY v0.3.0^22^. The remaining high-quality reads were then ***de novo*** assembled with NextDenovo v2.5.0^23^. This initial assembly was corrected twice using Nanopore reads with the assistance of Racon v1.4.11^24^, followed by two additional rounds of correction using BGI reads with Pilon v1.23^25^. Duplicates were removed from the corrected genome using purge_dups v1.4^26^, resulting in a draft genome size of 2.55 Gb, ready for further scaffolding, annotation, and analysis as detailed in Table 1.

**Table 1:** The statistics of assembly result.

Item	Value
Total length (bp)	2,553,726,437
Total length without N (bp)	2,553,598,837
Total number	1,860
GC content (%)	39
N50 (bp)	93,378,093
N90 (bp)	1,993,458
Average (bp)	4,372,819.24
Median (bp)	420,928.00
Min (bp)	5,000
Max (bp)	135,182,039

**Fig. 4:**
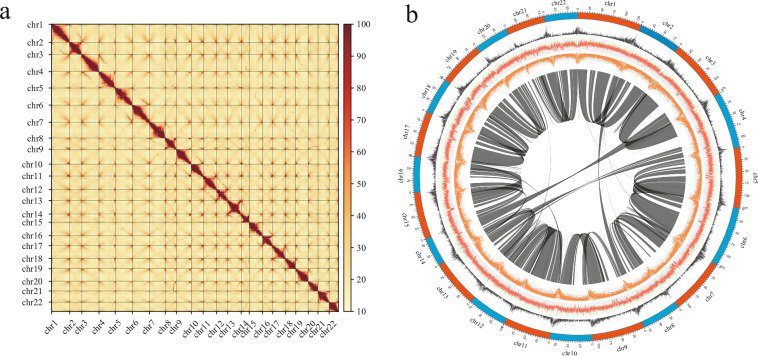
Interchromosomal Hi-C contact map (**a**) and Chromosomes circle (**b**) of *C. macrophyllum* genome. The circle diagram depicts the following from outer to inner layers: 22 chromosomes, gene density, GC content, repeat density, genome collinearity.

Hi-C reads were first quality-filtered with fastp v0.21.0^19^ to remove low-quality bases and other contaminants. The cleaned pairs were aligned to the draft genome with HICUP v0.8.0^27^, and uniquely mapped reads were passed to ALLHiC v0.9.8^28^ to cluster, order, and orient scaffolds into pseudo-chromosomes. Hi-C contact matrices were converted to binary (.hic) format with 3D-DNA v180419^29^ and Juicer v1.6^29^; the resulting scaffolds were then visualised and manually curated in Juicebox v1.11.08^30^. In total, 1,298 contigs were anchored onto 22 pseudo-chromosomes, representing 83.70% of the assembled genome (Fig. 4a,b). The assembled chromosomes range from 60,630,240 bp to 135,182,039 bp in length (Table 2).

**Table 2:** The chromosome length of *C. macrophyllum*.

Chromosome	Length(bp)	contig number
chr1	135,182,039	86
chr2	89,935,979	64
chr3	128,201,129	107
chr4	120,690,979	67
chr5	127,734,635	88
chr6	127,512,024	85
chr7	117,834,486	84
chr8	82,476,535	46
chr9	111,504,340	55
chr10	88,254,012	48
chr11	100,153,730	60
chr12	82,865,333	34
chr13	99,986,224	59
chr14	60,630,240	30
chr15	99,008,229	79
chr16	75,321,247	39
chr17	95,698,442	57
chr18	75,896,994	55
chr19	93,378,093	40
chr20	72,331,503	24
chr21	82,863,201	48
chr22	69,979,481	43
chrUnn	416,287,562	563

### Genome prediction and annotation

Repetitive elements in the *C. macrophyllum* genome were annotated with a pipeline that integrated homology-based and ***de novo*** strategies. An initial repeat library was constructed using LTR_FINDER v1.0.7^31^, LTRharvest v1.62^32^, and RepeatModeler v2.0.4^33^. Unidentified sequences were typed with TEclass v2.1.3^34^ and merged with Repbase v20181026^35^ database to yield the final library. This library was then used by RepeatMasker v4.1.5^36^ to mask repetitive sequences within the genome and by RepeatProteinMask v4.1.5 (https://github.com/Dfam-consortium/RepeatMasker) to predict repeat sequences based on TE protein types. Tandem repeat sequences were identified using Tandem Repeats Finder v4.09^37^ and MISA v2.1^38^. Comprehensive analysis revealed that repetitive elements comprised 2.13 Gb, or 83.23% of the total genome size (Table 3). Of this, interspersed repeats accounted for 1.78 Gb, or 69.69% of the genome, while tandem repeats occupied 345.84 Mb, representing 13.54% of the genome. Within the interspersed repeats category, DNA transposons constituted 13.41% of the genome, Long Interspersed Elements (LINEs) accounted for 4.67%, Short Interspersed Elements (SINEs) contributed 0.10%, and Long Terminal Repeat retrotransposons (LTRs) made up 64.76%. Among the LTR retrotransposons, LTR-Gypsy elements were the most prevalent, representing 30.14%, followed by LTR-Copia elements at 16.13% (Table 3).

**Table 3:** Statistics of repeat sequences in *C. macrophyllum* genome.

Type	Length (bp)	Percentage of genome (%)
DNA	342,578,348	13.41
LINE	119,315,477	4.67
SINE	2,493,817	0.1
LTR	1,653,702,602	64.76
LTR-Gypsy	769,568,716	30.14
LTR-Copia	412,034,617	16.13
Other	3,158	0
Unknown	118,258,723	4.63
Tandem repeats	345,844,520	13.543
Total	2,125,457,799	83.23

Non-coding RNA, which lack protein-coding potential, was predicted through various approaches. For tRNA prediction, tRNAscan-SE v2.0.12^39^ was utilized, while rRNA prediction was performed using RNAmmer v1.2^40^. To identify ncRNA, including snRNA and miRNA, INFERNAL v1.1.4^41^ was applied, referencing the Rfam database. As a result, our annotation process identified a total of 316 miRNAs, 2,768 tRNAs, 2,348 rRNAs, and 1,467 snRNAs in the *C. macrophyllum* genome (Table 4).

**Table 4:** The result of non-coding RNA annotation of the *C. macrophyllum* genome.

Type		Copy number	Average length(bp)	Total length(bp)	Percentage of genome (%)
miRNA		316	116	36788	0.0014
tRNA		2768	75	208949	0.0082
rRNA	rRNA	2348	364	855299	0.0335
	18S	83	1805	149819	0.0059
	28S	91	4945	450027	0.0176
	5.8S	885	117	103339	0.004
	5S	1289	118	152114	0.006
snRNA	snRNA	1467	120	175898	0.0069
	CD-box	970	111	107975	0.0042
	HACA-box	120	132	15786	0.0006
	splicing	377	138	52137	0.002
	scaRNA	0	0	0	0

The genome structure of *C. macrophyllum* was inferred through an integrative approach that combined **ab initio**, homology-based, and transcriptome-based predictions. For **Ab initio** prediction, Augustus v3.5.0^42^ and GlimmerHMM v3.0.4^43^ were applied. For homology-based prediction, protein sequences from *Chrysosplenium sinicum*, *Kalanchoe fedtschenkoi*, *Kalanchoe laxiflora*, *Rhodiola crenulata*, and *Arabidopsis thaliana*, were aligned to the the *C. macrophyllum* genome using tblastn v2.13.0^44^, after which transcript and protein-coding region were refined with Exonerate v2.4.0^45^. Transcriptome-based prediction combined BGI short reads and ONT full-length reads. Filtered BGI reads were mapped with HISAT2 v2.2.1^46^ and assembled with StringTie v2.2.1^47^. ONT reads were filtered using NanoFilt v2.8.0^48^ and identified via Pychopper v2.7.5 (https://github.com/epi2me-labs/pychopper). The resulting sequences were aligned to the *C. macrophyllum* genome using minimap2 v2.26-r1175^49^, and the resulting BAM files were reconstructed into transcripts using StringTie v2.2.1^47^. The resulting assemblies were merged with TAMA v1.0^50^, and open reading frames were identified using TransDecoder v5.7.0 (https://github.com/TransDecoder/TransDecoder). Finally, MAKER v3.01.03^51^ integrated the evidence from all three approaches to yield the consensus gene set. The resulting annotation comprises 62,921 protein-coding genes, with mean gene and CDS lengths of 4,086 bp and 1,123 bp, respectively; genes contain an average of 4.76 exons, with mean exon and intron lengths of 302 bp and 702 bp (Table 5).

**Table 5:** Statistics of protein-coding genes in *C. macrophyllum* genome.

Method	Software	Species	Gene number	Average gene length (bp)	Average CDS length (bp)	Average exon per gene	Average exon length (bp)	Average intron length (bp)
Ab initio	GlimmmerHMM		150,436	10,952.24	857.51	2.7	317.97	5,949.18
	AUGUSTUS		66,466	3,219.44	1,796.38	5.34	336.58	328.11
Homology	Exonerate	*Arabidopsis thaliana*	103,338	9,369.66	761.62	3.12	243.8	4,052.80
		*Chrysosplenium sinicum*	193,644	20,177.13	768.99	2.65	289.66	11,728.47
		*Kalanchoe fedtschenkoi*	112,888	7,579.61	717.39	2.97	241.55	3,483.49
		*Kalanchoe laxiflora*	117,964	8,271.90	722.07	2.95	244.66	3,869.03
		*Rhodiola crenulata*	146,034	20,869.55	774.2	2.6	297.86	12,566.12
RNAseq	TransDecoder		36,897	6,218.27	955.74	6.37	441.03	634.69
Integration	Maker		38,930	6,033.53	1,139.71	6.09	236.5	902.62
Final set	Anno-self		62,921	4,086.31	1,122.66	4.76	302.17	702.4

Predicted protein sequences were compared with the UniProt and NCBI non-redundant (NR) databases using DIAMOND v2.1.8^52^ to obtain high-confidence homologues. Conserved motifs and domains were identified with InterProScan v5.55–88.0^53^, and complementary domain searches were carried out with HMMER v3.3.2^54^. Gene Ontology (GO) terms were assigned by merging DIAMOND hits with InterPro-derived GO mappings in Blast2GO v4.1^55^, and GO terms were subsequently linked to their corresponding Enzyme Commission (EC) numbers. Kyoto Encyclopedia of Genes and Genomes (KEGG) orthologues were predicted via the KAAS (https://www.genome.jp/kegg/kaas/) web server. Overall, 58,939 genes —representing 93.67% of the predicted protein-coding set —received functional annotation in at least one database, and 1,319 genes were annotated across all databases (Table 6).

**Table 6:** The result of function annotation of the *C. macrophyllum* genome.

Type	Database	Count	Percentage of genome (%)
All		62,921	
Annotated	KEGG	25,657	40.78
	Pathway	14,021	22.28
	Nr	55,251	87.81
	Uniprot	55,731	88.57
	GO	40,542	64.43
	KOG	4,615	7.33
	Pfam	43,842	69.68
	Interpro	57,744	91.77
	Total annotated	58,939	93.67
Unannotated		3,982	6.33

## Data Records

The raw sequencing data used for genome assembly and annotation have been deposited in the National Genomics Data Center (NGDC)^56,57^, Beijing Institute of Genomics, Chinese Academy of Sciences/China National Center for Bioinformation, under the BioProject accession number PRJCA025550, and are publicly accessible at https://ngdc.cncb.ac.cn/bioproject. BGI short-reads, Oxford Nanopore reads, Hi-C reads, and RNA-seq data have been deposited in the Genome Sequence Archive^58^ in NGDC under the accession number CRR1136209^59^, CRR1136208^60^, CRR1136210^61^/CRR1136211^62^, and CRR1136212^63^/CRR1136213^64^, respectively. The chromosomal-level genome assembly data have been stored in GenBank with the accession number JBISEJ000000000^65^. Additionally, the genome annotation file is available in Figshare^66^.

## Technical Validation

To evaluate the accuracy and completeness of the *C. macrophyllum* genome, two complementary strategies were applied. Quality-filtered BGI short reads were first remapped to the assembly with BWA v5.3.0^67^, and 97.94% of reads aligned, indicating high alignment efficiency. Genome completeness was then evaluated with Benchmarking Universal Single-Copy Orthologs (BUSCO v5.3.0)^68^ based on the embryophyta_odb10 reference set (1,614 conserved orthologues). The assembly contained 98.6% complete genes, of which 29.6% were single-copy and 69.1% were duplicated; only 0.6% were fragmented and 0.7% were missing (Table 7). An identical BUSCO analysis of the annotated gene set recovered 1,592 complete genes (98.6% completeness), comprising 656 single-copy (40.6%) and 936 duplicated (58.0%) genes (Table 7). Taken together, these results confirm that both the genome assembly and its annotation are highly complete and reliable.

**Table 7:** BUSCO analysis of *C. macrophyllum* genome.

Item	Assembly		Annotation	
	Proteins	Percentage (%)	Proteins	Percentage (%)
Complete BUSCOs	1594	98.7	1592	98.6
Complete Single-Copy BUSCOs	478	29.6	656	40.6
Complete Duplicated BUSCOs	1116	69.1	936	58
Fragmented BUSCOs	9	0.6	9	0.6
Missing BUSCOs	11	0.7	13	0.8
Total BUSCO groups searched	1614	100	1614	100

## Data Availability

All data processing commands and pipelines were executed in accordance with the instructions and guidelines provided by the respective bioinformatic software. No custom scripts or code were used in this study.
